# Is gastroenterology research in decline? A comparison of abstract publication rates from The British Society of Gastroenterology meetings between 1995 and 2005

**DOI:** 10.12688/f1000research.2-59.v1

**Published:** 2013-02-22

**Authors:** Sarah Prendergast, Katharina Mattishent, Tom Broughton, Ian Beales

**Affiliations:** 1 Norwich Medical School, University of East Anglia, Norwich, NR4 7TJ, UK; 2 Department of Gastroenterology, Norfolk and Norwich University Hospitals NHS Foundation Trust, Norwich, NR4 7UY, UK

## Abstract

**Background: **Reports have suggested that academic medicine may be in decline within the UK. Further evidence suggests that rates of subsequent full publication of abstracts presented at major scientific meetings are low and may be declining. We have compared the publication rates of abstracts presented at meetings of the British Society of Gastroenterology (BSG) between 1995 and 2005 and examined factors associated with full paper publication.

**Methods:** Abstracts presented at BSG meetings in 1995 and 2005 were assessed by cross-referencing with multiple databases. Abstract characteristics associated with publication were analysed.

**Results: **There were no differences in overall publication rates, impact factors or time to publication between 1995 and 2005. Overall, basic-science abstracts were twice as likely to achieve full publication than non-basic science. There was a significant fall in the publication rates for case series and audits, and significantly increased rates for fundamental/basic-science abstracts over the study period. There were non-significant increases in publication rates for controlled trials and systematic reviews. In general, publication rates for all predominantly clinically orientated abstracts reduced between the two periods with the most notable fall occurring in nutrition.

**Conclusions:** There was no evidence of a decline in overall abstract publication rates between 1995 and 2005. There seemed to be trend for increased publication rates of abstracts using perceived high-quality study methodologies with a corresponding decrease in those with lower quality methods. The proportion of basic-science abstracts is likely to be a determinant of overall full publication rates following scientific meetings.

## Background

Abstracts are presented at national and international conferences to rapidly communicate the results of new and original research. This process also allows the researcher to receive preliminary and informal peer review from fellow researchers in the field. This may be an important part of academic development, as it helps authors to identify potential errors and to develop alternative interpretations of their results before proceeding to submission for full publication. It has been suggested that although abstracts submitted to conferences are peer-reviewed; this process may not be as rigorous as that of an indexed journal considering full publication ^1^.

Acceptance of an abstract for presentation at a conference may imply to the researcher that full publication is likely. Presentation of an abstract is certainly a positive sign that publication may be more likely, but this is not certain ^2^. Journal publication rates of submitted abstracts vary widely between 11% and 78% for different medical specialties ^3– 7^. A Cochrane review examined abstract publication rates of all medical subspecialties and reported a mean full (peer-reviwed journal) publication rate of 44.5%, with higher rates of 63.1% for randomised or controlled clinical trials ^5^.

Criteria for the acceptance of abstracts for conference presentation include such factors as: evaluation of whether the ideas presented are new, potentially significant, interesting and plausible. However, at the time of presentation, there may be insufficient information concerning data or methodology to assess the true scientific value of the research ^1^. Inherently this acceptance process is inevitably not as rigorous as journal peer-review.

Reasons for subsequent non-publication are not entirely clear. Four main reasons have been suggested: (1) lack of time to prepare a manuscript for publication, (2) the study may be still on-going, (3) relationships with co-authors sometimes present a barrier to final publication, and (4) authors’ feelings that pursuit of publication is a low priority ^8^. To these, the possibility of the study being of low scientific quality must be added. It has been suggested that improved preparation prior to the study and stricter guidelines to limit the presentation of abstracts at national and international meetings may help to reduce the number of unpublished studies ^8^.

The percentage of abstracts that are eventually published is a potentially important clinical issue. Abstract presentations may be deemed to be more credible than is justifiable. For example, The American Academy of Orthopaedic Surgeons suggested that many national meeting attendees may alter their surgical practices, based on informally acquired and unscrutinsed information obtained from abstracts ^6^. This practice has important implications for the development of evidence-based medicine. In addition, cardiology abstracts have been cited as references in journal reference lists, with no difference in citation rates between published and un-published papers ^9^. These findings emphasise the need for caution and restraint in citing abstracts as full references. Other notable sources have discouraged the citation of abstracts, on the grounds that such practices may propagate invalid and erroneous conclusions ^1^. In support of these concerns, many journals will not accept abstract citations ^10^. Clearly, the citing of material that has not been subjected to the full rigour of peer-review is inadvisable and may undermine the scientific value of the work.

An important factor that appears to influence whether a study described in an abstract proceeds to full publication is the presence of ‘positive’ results. This refers to results that are significant in favour of the experimental treatment ^11^. In a study of research abstracts at an emergency medicine meeting, positive-outcome bias was evident. In addition publication did not relate to study design or quality ^2^. Other factors associated with full publication include oral presentation, acceptance for meeting presentation, randomised controlled study design, and controlled clinical trials and basic research ^5^.

It also appears that the length of time taken between the presentation of the abstract and full publication is influenced by this so-called ‘publication bias’. The Cochrane review examined the time to publication of a cohort of 196 clinical trials. The investigators found that just over half the trials were published, but that those with positive results (i.e. statistically significant in favour of the experimental arm) were published within 4 or 5 years, whereas those with null or negative results (i.e. not statistically significant or statistically significant in favour of the control arm) were published after 6–8 years ^12^. It has been suggested that these findings had important implications because if trials with positive findings are stopped earlier and published quicker than those with negative findings, then new treatments might be mistakenly assumed to be effective ^12^. These findings also have important implications for the timing of the initiation and updating of a systematic review. The earlier publication of positive results, combined with the tendency to publish ‘significant’ results means that systematic reviews will tend to over-estimate treatment effects ^5^.

It has also been noted that study type may be an important factor in determining whether a study is published. One study of two UK national paediatric meetings found that most randomised controlled trials were published, but that observational studies submitted were published less often ^13^. Sources have suggested that, because many factors important in a scientific study are impossible to control in a retrospective study, it is not surprising that prospective studies are published at much higher rates than retrospective studies ^1^. Thus the recommendation of well-designed prospective studies with high statistical power should be encouraged.

Recent reports have suggested that academic medicine in the UK may be in decline and that research output is reducing ^14, 15^. Possible reasons for this development include the perceived overwhelming bureaucratic processes of research governance ^16^ and lack of funding ^15, 16^. This concept is supported by evidence that between 1994 and 2002 the number of publications published by gastroenterology specialist registrar trainees before starting consultant posts, had fallen significantly from a median of 19 in 1993 to a median of 5 by 2002 ^17^.

Further supporting evidence includes a downward trend for the percentage of abstract presented at the British Society of Gastroenterology (BSG) annual meetings to reach full publication in the years after 1994. The authors inferred that this observation could be taken as a surrogate marker for a decline in research activity within the UK gastroenterology community, but this study did not further examine whether any study type or subject was more or less likely to reach full publication. They recommended that further work was required to validate their findings ^18^.

Therefore, there appear to be considerable uncertainties as to whether academic medicine and research activity within the United Kingdom's health service are declining and there are very little data concerning predictors of subsequent full publication from abstracts particularly related to gastroenterology, although this latter question may well be more generic. In addition, presenting abstracts at meetings is generally viewed as contributing to career progression for post-qualification doctors in training and presentation of abstracts is used in the scoring of applicants for training posts. There are little data to guide trainees or trainers on how valuable these activities are. In this study we have examined the publication rates from two BSG meetings separted by 10 years to determine if publication rates have changed and also to examine whether there was any relationship between study type, subspeciality within gastroenterology and time-lag to publication.

## Methods

Hard copies of abstracts presented at the BSG scientific meetings were obtained for the years 1995 and 2005. There were two separate BSG meetings in 1995 and one single conference in 2005; data for both meetings in 1995 were combined. Data collection was performed retrospectively starting in 2010, ensuring that a period of at least four years had elapsed since the last meeting chosen to be assessed. This approach was taken to align this study with previous work in the field ^18^ and because it has been reported that the upper time limit to publication after an abstract is presented at a meeting is four years ^3, 4, 9^. A further repeat search was performed in September 2012 to check for any very delayed publications.

All abstracts presented at the BSG conference for 1995 and 2005 were collected and cross-referenced with the Ovid, Medline, EMBASE, The Cochrane Library, Web of Science and Wiley Interscience databases to assess for evidence of full publication.

Abstracts were cross referenced using first-author, senior author and at least one key word from the abstract title. Where an abstract appeared to have been published, abstracts and published articles were further examined to ensure that they represented the same study. If two or more abstracts were part of single fully published manuscript, then each one was counted individually as a publication for all aspects of the study.

In addition, study type, subspeciality within gastroenterology, type of study, citation, impact factor and lag- time to publication were also assessed. The impact factor of the journals for the year in which the paper was published was determined using the Web of Science database. Abstracts from each year were assessed independently by two of the authors; any differences were resolved by consensus under the direction of the senior author (ILPB).

Publication rates and odds ratios with 95% confidence intervals were used to compare abstracts presented in 1995 and 2005 using chi-squared test. Impact factors and time to publication were calculated as mean ± standard deviation and compared using Students t-test. Bonferroni correction was applied for multiple comparisons.

## Results

From the meetings in the 2 years, a total of 938 records were analysed; all records were complete and no records were excluded due to anomalies or missing data. The number of abstracts presented over the two meetings in 1995 was 497, and 441 were presented at the at the single 2005 conference. The number of abstracts which proceeded to full publication was remarkably similar in the 2 years, 88 (17.7%) in 1995 and 77 (17.4%) in 2005; odds ratio (OR) for publication in 2005 compared to 1995 (0.98, 95% CI 0.79 – 1.39).

We next examined whether study type or overall topic seemed to be associated with publication rates. The data are summarised in Table 1. The most striking changes were a significant fall in the publication rates for case series (OR 0.47, 0.26 – 0.85) and audit articles (with no publications at all from 2005), coupled with a significant increase in publication rates for basic/fundamental science between the 2 years. In light of the latter, the overall chance of publication fell for predominantly “clinical“ (non-basic science) abstracts between 1995 and 2005, (OR 0.74, 0.48 – 1.19). In fact, including data from both years, abstracts focused on basic or fundamental science were more than twice as likely to achieve full publication than non-basic-science abstracts (OR 2.19, 1.51 – 3.16).

**Table 1.** Odds ratios with 95% confidence intervals for full publication of abstracts presented in 2005 compared to those presented in 1995.

	Odds ratio of publication in 2005 compared to 1995 (95% CI)	
All	0.98 (0.79 – 1.39)	
Randomized controlled trial	1.68 (0.38 – 7.60)	
Systematic review/metanalysis	1.69 (0.10 – 58.5)	
Case series	0.47 (0.26 – 0.85)	*P* < 0.05
Audit	0.0 (0.0 – 2.42)	
Service evaluation	1.58 (0.27 – 12.73)	
Epidemiology	0.72 (0.27 – 1.87)	
Fundamental science	2.15 (1.13 – 4.05)	*P* < 0.05
Endoscopy	0.69 (0.30 – 1.59)	
Surgery	0.42 (0.11 – 1.38)	
Oncology	0.59 (0.19 – 1.88)	
Inflammatory bowel disease	0.48 (0.19 – 1.22)	
Liver disease	0.66 (0.29 – 1.51)	
Nutrition	0.26 (0.01 – 0.97)	*P* < 0.05
Genetics	2.17 (0.50 – 9.55)	
Cell biology	1.85 (0.41 – 8.35)	
Pharmacology	2.29 (1.02 – 14.15)	
Physiology	2.24 (0.45 – 11.08)	
Immunology	1.87 (0.39 – 9.21)	

We further examined whether any major area was responsible for the increased publication rates in fundamental science: publication rates increased in all the major areas examined, the greatest rise being in pharmacology but overall rates increased in all areas.

Within the major subspecialties of gastroenterology, rates of publication generally fell in all areas, the greatest and most significant fall being in nutrition (OR 0.26, 0.01 – 0.97).

The mean impact factor of the journals that the abstracts were published in from 1995 was 4.67 (± 3.41). The mean impact factor for 2005 was 3.87 (± 2.43). This difference was not statistically significant. The mean time to publication in 1995 was 22.2 months (± 16.9), this was slightly faster for 2005 (mean time 18.6 ± 13.3), but not statistically different.

## Discussion

This study found no significant difference in the percentage of abstracts presented at the BSG conferences in 1995 compared with 2005, that subsequently achieved full publication. These findings are at variance with some earlier studies ^17, 18^ that indicated that the abstract publication rates were declining within gastroenterology. The overall publication rate in 1995 was relatively low and it is possible that any decline had already plateaued by 1995, or else 1995 was an outlier year with a low publication rate. Further analysis of surrounding years will be required to address this.

Despite the overall similarities in publication rates between the two different years, 10 years apart, several interesting differences were seen. There was a marked decline in subsequent publication of case series and audit articles, but this was balanced by an increase in publication rates for fundamental science. Overall publication rates did fall for predominantly clinical studies.

There are two main conclusions that can be drawn from these changes. Firstly that there does seem to be reduction in the rate of full publication of perceived “lower quality” research (case series and audit articles) with an increase in publication rates for basic science, which is usually seen as “higher” quality because of the much greater control of conditions and experimental planning possible ^19^. This trend would seem to be in keeping with increased editorial rigour in peer-reviewed publications. There were only a small number of randomised trials in the dataset but the upward trend for publication rates for both these and systematic reviews over the study period would seem consistent with this concept ^19^. Another factor to be considered is that it is likely that in a greater proportion of basic-science abstracts, the lead author would have been a “professional” scientist (possibly without medical training), whereas “clinical” abstracts would more likely be produced by trainees or consultants in clinical posts. We can speculate that the greater time and focus available, fewer competing clinical commitments and less movement of abstract authors between training posts and regions exhibited by basic-science abstract authors all contributed to the increased publication rate.

The second conclusion is that crude publication rates are probably an insufficient tool to analyse abstract publication rates from a broadly based scientific meeting such as the BSG annual meetings, which genuinely spans bench to bedside to community, as the proportion of underlying basic-science abstracts may significantly influence publication rates. Further studies comparing publication rates with similar meetings such as cardiology or respiratory societies or basic-science-focused meetings such as the Physiological Society would be interesting.

Within basic science, there did not seem to be a predominant area that led the increased publication rates; there seemed to be an overall increase. We accept that classification within these areas can be difficult due to considerable overlap between focus and experimental techniques within the fundamental science themes.

There was a general decline in publication rates in all major subspecialty areas of clinical gastroenterology and hepatology: this probably reflects a general trend for reduced publication rates for primarily clinical studies rather than a shift influenced by topicality or fashion for certain areas within gastroenterology between 1995 and 2005. The most marked fall in publication rates occurred in nutrition, this despite nutritional science and clinical nutrition having a much higher profile over the latter years of the study period. One explanation for this may be that although the BSG has an active nutrition section, there are other scientific meetings and societies, particularly the multidisciplinary British Association for Parenteral and Enteral Nutrition (BAPEN) and perhaps the higher quality nutrition papers from 2005 were presented as such meetings instead of the BSG.

It has been suggested that certain features of abstracts, such as sample size, study type, methodology and the presence of statistically significant results may influence the chances of full publication ^20^. A fuller examination of the published papers than was possible in this study might reveal more information on these factors. We have considered whether the differences in the number of BSG conferences in 1995 compared with 2005 may have had any effect on the results but feel that this was unlikely and therefore was not pursued further.

Overall publication rates were low, at approximately 17%, but these fall well within the ranges reported from other major meetings ^3– 5^ and it must be appreciated that overall publication rates are not only influenced by the vigour with which authors pursue subsequent publication, but also the perhaps competing requirements of journal editors for high-quality, rigorous research papers and conference organisers wishing to maximise attendance at their meetings. Increasing the number of abstracts presented and hence the associated number of presenters is one such way of increasing attendance; theoretically this could lead to increased presentation of abstracts of a lower scientific quality.

The results obtained from this study suggest that there has not been a decline in abstract publication rates between the years 1995 and 2005. Where publication rates are seen as a surrogate marker for research activity ^18^, this suggests gastroenterology research activity has remained relatively stable, although as discussed above, the crude publication rate may be an inadequate tool to measure this. One other factor to be considered is that although the meetings considered are organised by the British Society of Gastroenterology, these are international meetings and our data cannot be used to specifically imply growth or decline in gastroenterology research activity specifically in the United Kingdom; we did not analyse abstracts for country or origin.

In conclusion, we have confirmed that abstracts presented at scientific meetings commonly do not result in subsequent full peer-reviewed publications and this should perhaps be considered when appraising the quality of studies published as abstracts only. Within gastroenterology we have shown a decline in publication of case series and audit articles, and an increase in publication of basic/fundamental science. Further studies defining the predictors of subsequent publication as well as comparisons with other meetings and subsequent years would be interesting.

## Supplementary data

Appendix 1. Individual abstract-level data for abstracts presented at the BSG meeting in 1995 and 2005. The dataset shows the extraction of data related to individual abstracts presented at the BSG meetings in 1995 and 2005. Classification of abstracts by type of study and topic area and details of subsequent peer-reviewed publications are shown.

Appendix 2. Unadjusted odds ratios (OR) for subsequent publication of abstracts presented to the British Society of Gastroenterology in 2005 compared to 1995. Table showing odds ratios for the subsequent publication in peer-reviewed journals of abstracts presented at the BSG meeting in 2005 compared to those presented at the 1995 meetings. Results are shown by classification of abstract by study type, main area of study and discipline for fundamental (life) sciences.
