# Supplementary material for: Is gastroenterology research in decline? A comparison of abstract publication rates from The British Society of Gastroenterology meetings between 1995 and 2005
Source: F1000Res. 2013 Feb 22;2:59. [Version 1] doi: 10.12688/f1000research.2-59.v1 (PMC3752733; doi:10.12688/f1000research.2-59.v1)
Supplement: Peer review history

# Peer review: Referee response for version 1

**Published:** 2013-03-04

**Authors:** Harriet Gordon

**Affiliations:** Hampshire Hospitals NHS Trust, Hampshire, UK

This is a valid and helpful study

I have read this submission. I believe that I have an appropriate level of expertise to confirm that it is of an acceptable scientific standard.

# Peer review: Referee response for version 1

**Published:** 2013-02-26

**Authors:** Jean Crabtree

**Affiliations:** Leeds Institute of Molecular Medicine, St James's University Hospital, Leeds, UK

The paper would be improved if the following were added:

- An inclusion in the methods section of the classification criteria for abstracts based on basic/fundamental science.
- On page 6, the authors state 'proportion of basic science abstracts may significantly influence publication rates'. Data on % abstracts in clinical, audit and basic/fundamental science areas in 1995 and 2005 should be included so changes in patterns of submission/acceptance over the 10 year period can be determined.
- Information on whether the journal impact factors of clinical versus fundamental/basic science publications differed.
- Are data available on the funding sources for abstracts published as full papers? Are abstracts of work funded by national research councils and charities more likely to be published than pharmaceutically funded studies?

I have read this submission. I believe that I have an appropriate level of expertise to confirm that it is of an acceptable scientific standard.

# Peer review: Referee response for version 1

**Published:** 2013-02-25

**Authors:** Penny Neild

**Affiliations:** St. George's Hospital, London, UK

I have read this submission. I believe that I have an appropriate level of expertise to confirm that it is of an acceptable scientific standard.
